# Internalization of the Extracellular Full-Length Tau Inside Neuro2A and Cortical Cells Is Enhanced by Phosphorylation

**DOI:** 10.3390/biom6030036

**Published:** 2016-08-19

**Authors:** Mathilde Wauters, Ruddy Wattiez, Laurence Ris

**Affiliations:** 1 Department of Neuroscience, Research Institute for Biosciences, University of Mons, Mons 7000, Belgium; mathilde.wauters@umons.ac.be; 2 Department of Proteomics and Microbiology, Research Institute for Biosciences, University of Mons, Mons 7000, Belgium; ruddy.wattiez@umons.ac.be

**Keywords:** Tau, phosphorylation, internalization, spreading

## Abstract

Tau protein is mainly intracellular. However, several studies have demonstrated that full-length Tau can be released into the interstitial fluid of the brain. The physiological or pathological function of this extracellular Tau remains unknown. Moreover, as evidence suggests, extracellular Tau aggregates can be internalized by neurons, seeding Tau aggregation. However, much less is known about small species of Tau. In this study, we hypothesized that the status of phosphorylation could alter the internalization of recombinant Tau in Neuro2A and cortical cells. Our preliminary results revealed that the highly phosphorylated form of Tau entered the cells ten times more easily than a low phosphorylated one. This suggests that hyperphosphorylated Tau protein could spread between neurons in pathological conditions such as Alzheimer’s disease.

## 1. Introduction

The Tau protein is being increasingly studied because of its implication in a large number of disorders known as tauopathies, including Alzheimer’s disease (AD) [1]. It has been established that abnormal hyperphosphorylated Tau aggregates follow a stereotypical transmission pattern between connected regions of the brain during the course of AD, indicating a synaptic transmission of the pathology [2,3,4,5]. On the other hand, the hypothesis of a prion-like propagation is supported by the fact that aggregates can be internalized and then released by several pathways into the extracellular fluid from where they are able to transmit the pathological conformation to other cells [6,7]. This possibility is also supported by detection of aggregates in patients’ cerebrospinal fluid [8]. Taken together, these findings explain why Tau constitutes a particularly promising subject of study in this field.

Interestingly, more recent studies have focused on smaller species of Tau protein, such as monomers or oligomers, showing that they were also able to spread between neurons [9,10].

In this study, we focused on the status of phosphorylation of Tau protein in order to better understand its role in protein internalization. We found that small species of Tau, when applied extracellularly, were capable of entering Neuro2A cells, as demonstrated by Michel et al. [10], but also primary cortical cells. More importantly, phosphorylation of Tau promoted its internalization by 10-fold.

## 2. Results

### 2.1. Characterization of Recombinant Tau Proteins

**Figure 1:**
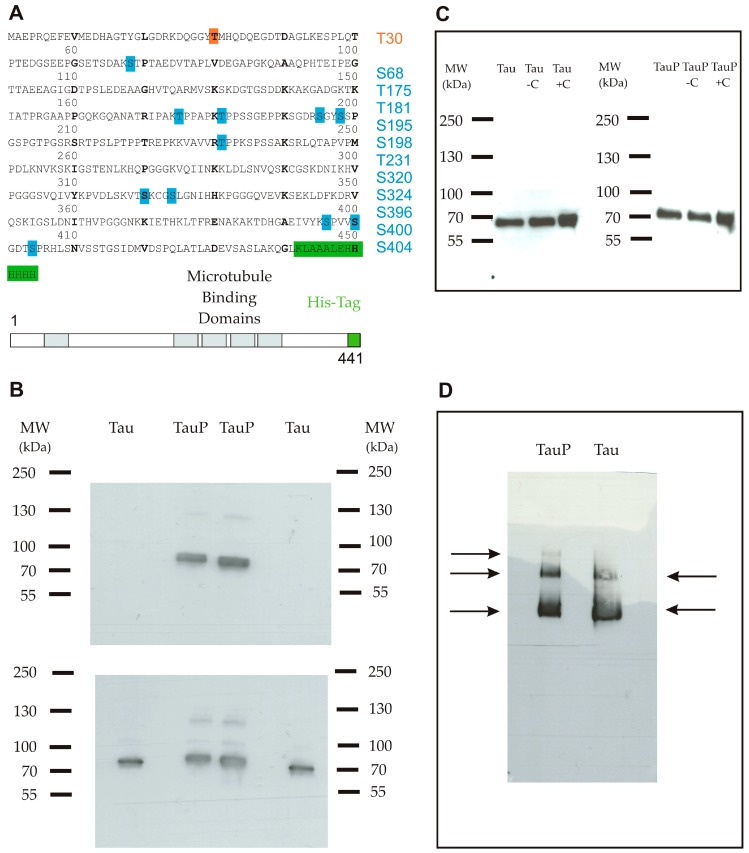
(**A**) Primary sequence of the full-length recombinant monomeric Tau protein 1-441 isoform (2N4R) coupled with a C-terminal His-tag (6× His). The protein comprised one phosphorylation site for the low phosphorylated form (orange) and 11 phosphorylation sites for the highly phosphorylated form (blue); (**B**) Western blot using an anti-phosphoT231 antibody. Upper panel shows a band only for the highly phosphorylated form. Lower panel is a control with an anti-Tau antibody and shows that both proteins were correctly loaded in duplicate; (**C**) Western blot using an anti-His-tag antibody shows that the Tau protein remained in a monomeric form in the purified stock (well 1), after an incubation of 6 h in medium only (well 2) and after an incubation of 6 h in the presence of cortical cells (**C**) (well 3) for low (left) and highly phosphorylated (right) Tau proteins; (**D**) Blue native polyacrylamide gel electrophoresis (BN-PAGE) followed by immunodetection using an anti-Tau antibody shows three bands for the highly phosphorylated form (from the top-down: trimers, dimers and monomers) and two bands for the low phosphorylated form (from the top-down: dimers and monomers).

Two forms of recombinant His-tagged Tau were compared in this study, one was glycogen synthase kinase 3 beta (GSK3β)-phosphorylated thanks to a co-transfection of Tau and GSK-3β proteins and the other one was not modified. In the text, we will refer to these two proteins as highly and low phosphorylated Tau proteins. The sequence and the phosphorylation state of the two proteins were verified by mass spectrometry (Figure 1A). The ratios of the signal intensities of phosphopeptide versus peptide were calculated to estimate the level of phosphorylation. For the highly phosphorylated Tau protein, only two phosphorylation sites of phosphopeptides possessed a non-phosphorylated version with a ratio of 10^5^/10^2^ for serine 198 and 2 × 10^5^/900 for threonine 181. In this form, 11 sites were identified as phosphorylated with a large amount of phosphopeptides justifying the term of highly phosphorylated used for this protein. Tau phosphorylation at threonine 231 was then assessed by western blot, which also supported a distinct phosphorylation state between both proteins (Figure 1B).

Western blot and blue native polyacrylamide gel electrophoresis (BN-PAGE) followed by immunodetection were performed as controls to ensure the aggregation state of purified Tau proteins. In western blot, only one band was apparent, suggesting that proteins remained in the monomeric form. A short shift was visible, deriving from the phosphate groups belonging to the highly phosphorylated Tau protein (Figure 1C). Three and two bands were detected in non-denaturing conditions, for highly and low phosphorylated Tau proteins respectively, suggesting the presence of a small proportion of dimers and trimers in addition to the principal monomeric form (Figure 1D). In neither case were oligomers or aggregates observed.

### 2.2. Recombinant Tau Proteins can Be Internalized by Neuro2A and Cortical Cells

**Figure 2:**
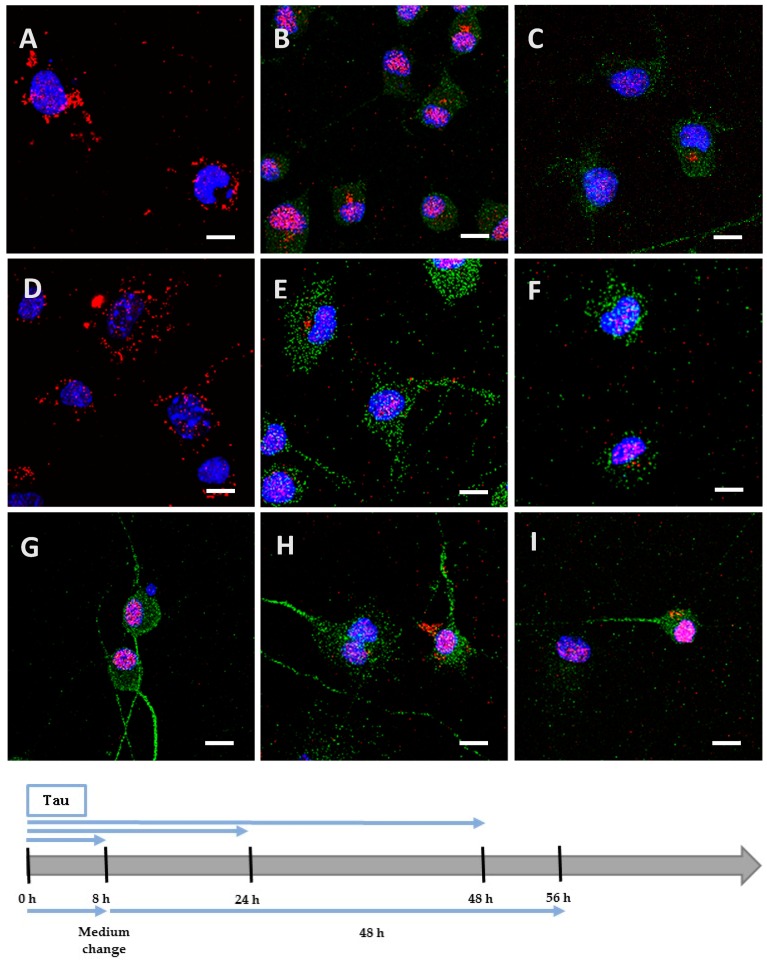
Internalization and processing of the Tau protein (15 µg; **A**–**C**,**H**) and its highly phosphorylated form (1.5 µg; **D**–**F**,**I**) by Neuro2A cells. Following 8 h (**A**,**D**), 24 h (**B**,**E**), or 48 h (**C**,**F**) of incubation with protein, cells were immunostained with a mouse anti-His-tag antibody targeted by an AlexaFluor^®^ 594 antibody (red) and a rabbit anti-microtubule-associated protein 2 (MAP2) antibody targeted by an AlexaFluor^®^ 488 antibody (green). In other experiments, cells were placed in contact with protein for 8 h, whereupon the medium was replaced by one devoid of protein and maintained for 48 h (**H**,**I**). The negative control (**G**) showed a non-specific distribution of the anti-His-tag antibody, localized only in the nucleus. Nuclei were stained with 4′,6-diamidino-2-phenylindole (DAPI). All scale bars correspond to 10 µm.

**Figure 3:**
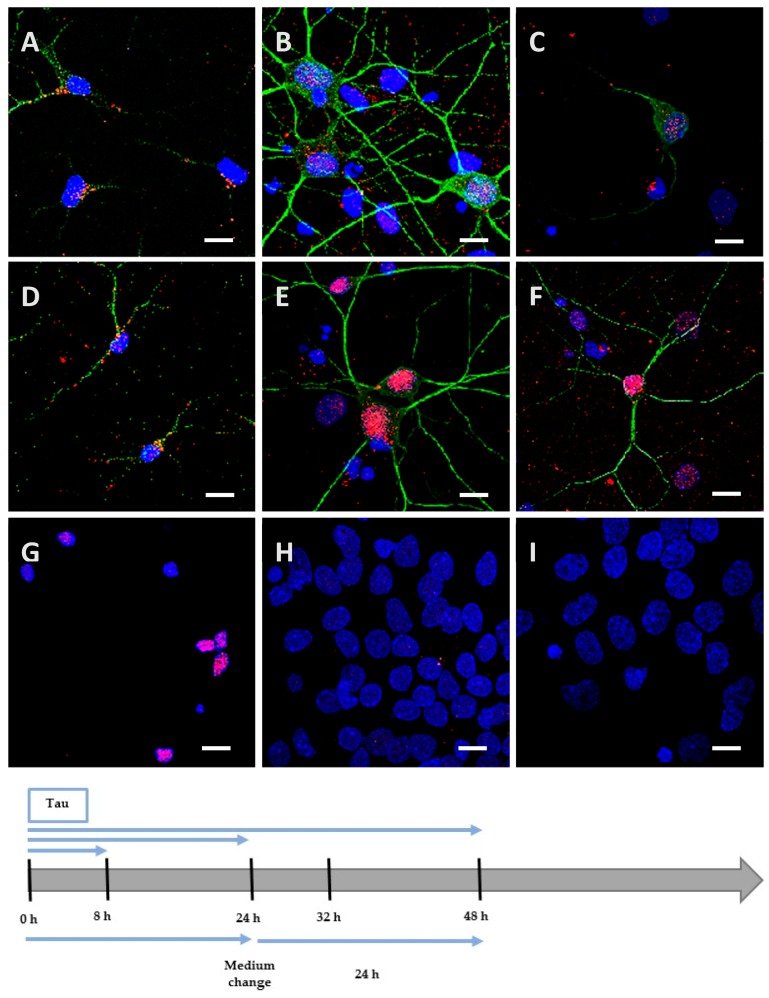
Internalization and processing of the Tau protein (15 µg; **A**–**C**,**H**) and its highly phosphorylated form (1.5 µg; **D**–**F**,**I**) by cortical cells. Following 8 h (**A**,**D**), 24 h (**B**,**E**), or 48 h (**C**,**F**) of incubation with protein, cells were immunostained with a mouse anti-His-tag antibody targeted by an AlexaFluor^®^ 594 antibody (red) and a rabbit anti-MAP2 antibody targeted by an AlexaFluor^®^ 488 antibody (green). In other experiments, cells were placed in contact with protein for 24 h, whereupon the medium was replaced by one devoid of protein and maintained for an additional 24 h (**H**,**I**). The negative control (**G**) showed a non-specific distribution of the anti-His-tag, localized only in the nucleus. Nuclei were stained with DAPI. The protein was sometimes detected outside cells where it was probably attached to the extracellular matrix. All scale bars correspond to 10 µm.

**Figure A1:**
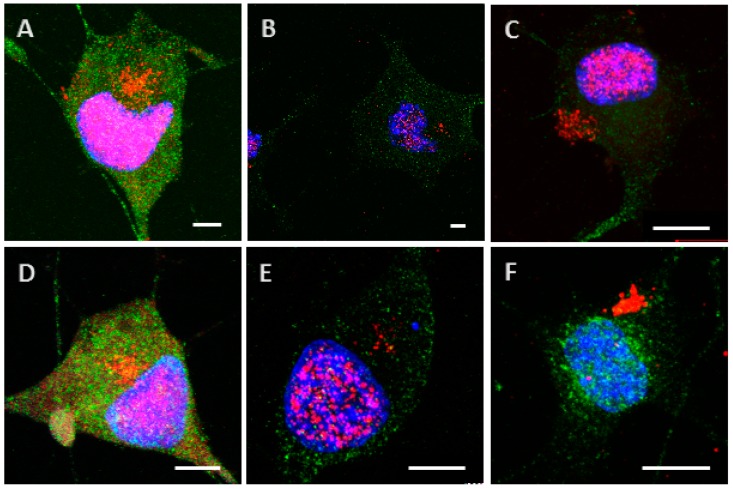
Internalization and processing of the Tau protein (15 µg; **A**–**C**) and its highly phosphorylated form (1.5 µg; **D**–**F**) by Neuro2A cells. Following 24 h (**A**,**D**), or 48 h (**B**,**E**) of incubation with protein, cells were immunostained with a mouse anti-His-tag antibody targeted by an AlexaFluor^®^ 594 antibody (red) and a rabbit anti-MAP2 antibody targeted by an AlexaFluor^®^ 488 antibody (green). In other experiments, cells were placed in contact with protein for 8 h, whereupon the medium was replaced by one devoid of protein and maintained for 48 h (**C**,**F**). Nuclei were stained with DAPI. Different digital zooms were applied to the 60× optical zoom: A: no zoom, B: 1.2×, C: 2×, D: 1.6×, E: 2.1×, F: 2.3×. All scale bars correspond to 5 µm.

**Figure A2:**
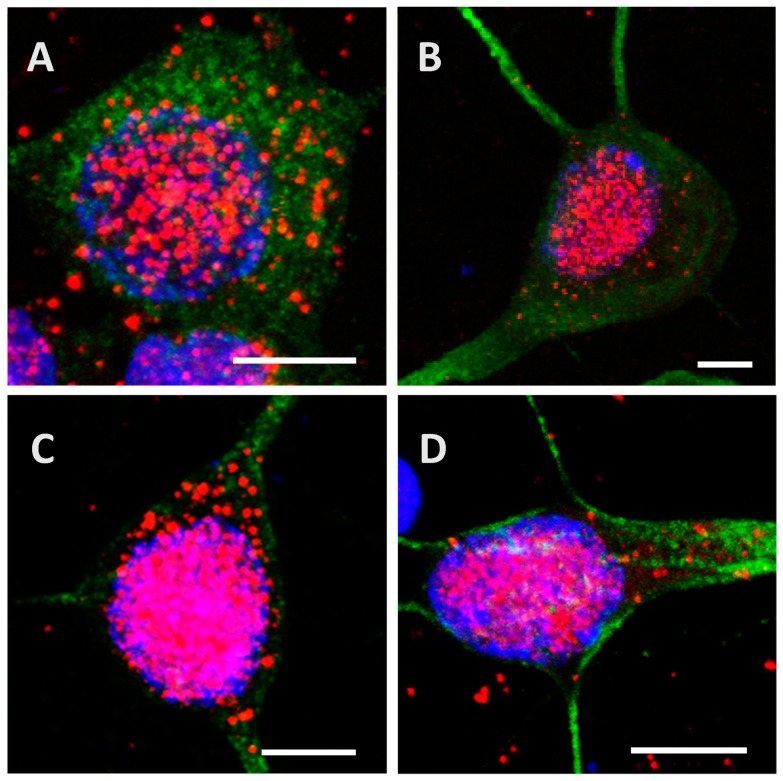
Internalization and processing of the Tau protein (15 µg; **A**,**B**) and its highly phosphorylated form (1.5 µg; **C**,**D**) by cortical cells. Following 24 h (**A**,**C**) or 48 h (**B**,**D**) of incubation with protein, cells were immunostained with a mouse anti-His-tag antibody targeted by an AlexaFluor^®^ 594 antibody (red) and with a rabbit anti-MAP2 antibody targeted by an AlexaFluor^®^ 488 antibody (green). Nuclei were stained with DAPI. The protein was sometimes detected outside cells where it was probably attached to the extracellular matrix. Different digital zooms were applied to the 60× optical zoom: A: 2.6×, B: 2.8×, C: 2.1×, D: 3×. All scale bars correspond to 5 µm.

**Figure A3:**
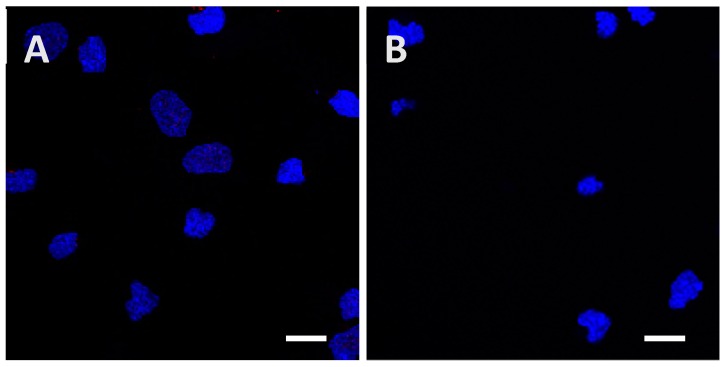
Determination of the minimal concentration needed to observe internalization of the low phosphorylated Tau protein inside cortical cells after 24 h of incubation. Initial protein amounts of 1.5 µg (**A**) and 4.5 µg (**B**) were used, but no signal was detected until 15 µg of the protein were added (Figure 2E and Figure 3E). Scale bars, 10 µm.

We incubated Neuro2A cells with the low phosphorylated Tau protein (15 µg at 1 µM concentration). After 8 h, the protein was mainly localized on the periphery of Neuro2A cells, although it had already begun to be internalized (Figure 2A), unlike the negative control (Figure 2G). After a longer incubation of 24 h (Figure 2B) and 48 h (Figure 2C), the protein was repeatedly observed gathering as inclusions near the nucleus, and neither spreading from the cytoplasm to the neurites, nor accumulating in the cytoplasm (See Figure A1 for images with digital zooms). The same conditions were applied to primary cortical cells, which presented identical results (Figure 3A–C) relative to the negative control (Figure 3G), except that the localization was more scattered inside the cells (See Figure A2 for images with digital zooms). Under the same experimental conditions, the application of 1.5 µg or 4.5 µg of low phosphorylated Tau protein did not lead to the detection of His-tagged protein inside the cells (Figure A3).

### 2.3. Phosphorylation Increases the Ability of the Protein to Enter Cells

**Figure A4:**
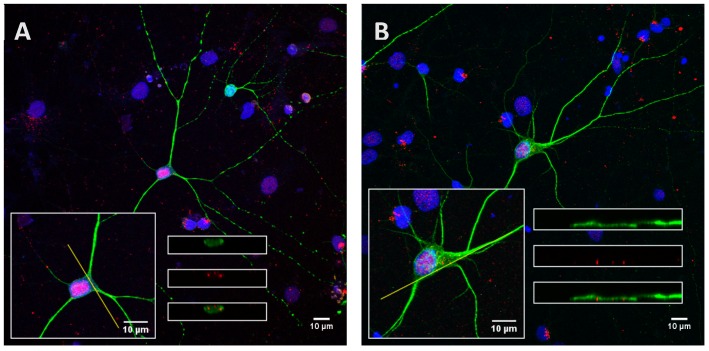
Orthogonal projections showing cross sections of the stacks along the yellow line. Cortical cells were incubated for 8 h with the Tau protein (15 µg; **A**) and its highly phosphorylated form (1.5 µg; **B**).

We hypothesized that, once highly phosphorylated, the protein would be more capable of penetrating the cells. Interestingly, after 8 h of incubation, the quantity (1.5 µg at 0.1 µM concentration) needed to detect the highly phosphorylated protein inside the cells was 10 times lower than the one needed to detect low phosphorylated protein. This result was observed both in Neuro2A (Figure 2D) and cortical cultures (Figure 3D). As controls, we verified that the anti-His-tag antibody had the same affinity for both proteins using an enzyme-linked immunosorbent assay (ELISA). The ratio of relative means of optical densities obtained for low and highly phosphorylated Tau proteins amounted to 0.99 after 10 min of incubation with 2,2′-azino-bis (3-ethylbenzothiazoline-6-sulphonic acid)-diammonium salt (ABTS)-H_2_O_2_. Beside the internalization properties, similar observations were made over time for the localization and spreading function of the culture model for the hyperphosphorylated form (Figure 2E,F and Figure 3E,F; Figure A1 and Figure A2). Once again, no accumulation was visible despite the continuous presence of the protein in the medium. The internalization of low and highly phosphorylated Tau protein inside cortical cells was also demonstrated by orthogonal projections of Z-stacked images (Figure A4).

### 2.4. The Processing of the Protein Depends on Cell Culture Models

**Figure A5:**
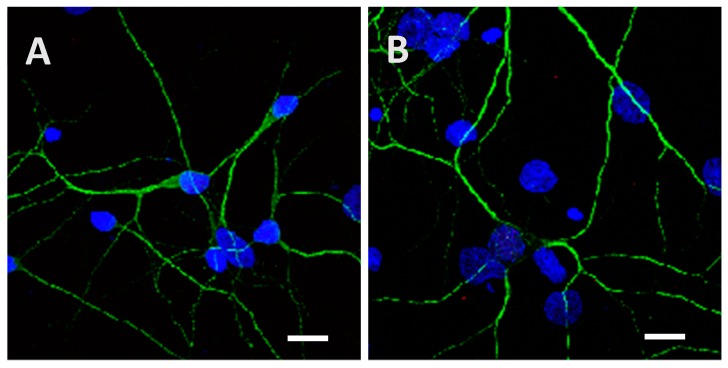
Low phosphorylated (15 µg; **A**) and highly phosphorylated Tau proteins (1.5 µg; **B**) were no longer observed inside cortical cells after an incubation of 8 h followed by a washing for 24 h with medium. Scale bars, 10 µm.

We then investigated the processing of the internalized protein by replacing the medium containing the cells with one devoid of protein. The medium containing the Neuro2A cells was changed after 8 h of incubation for a further 48 h. After this delay, low and highly phosphorylated proteins were still detected near the nucleus (Figure 2H,I). However, when cortical cells were incubated for 8 h (Figure A5) or 24 h with proteins (Figure 3H,I) before the medium was changed for a further 24 h, proteins were no longer detectable. This result could be explained by the degradation or the exocytosis of the proteins.

## 3. Discussion

While some studies have demonstrated that Tau monomers are not endocytosed by neural cells [7,11], the study performed by Michel et al. [10] shows that after 8 h, the monomeric wild-type Tau, at a concentration of 1 µm, is able to enter SH-SY5Y neuroblastoma cells. In the same way, Dujardin et al. [12] support the hypothesis that the wild type monomeric Tau is a candidate for explaining the propagation mechanism in sporadic tauopathies, both in vitro and in vivo. Interestingly, they specified that a synaptic transmission mainly occurred with the dephosphorylated form of the protein, which is inconsistent with our findings since we demonstrated a facilitated entrance of the Tau protein once it had been hyperphosphorylated. All these results tend to consolidate the evidence that monomeric Tau could play an important role in AD.

In this study, we used Neuro2A cells and an embryonic cortical cell culture to evaluate if different cell properties can modify Tau internalization and processing. Our observations differed in terms of distribution in the cytoplasm and processing of the protein, highlighting the importance of using an appropriate culture model. Despite similar internalization, protein was always detected inside Neuro2A cells after 2 days as inclusions near the nucleus, whereas it was scattered in cortical cells and disappeared after only 24 h. It still remains to be determined whether this absence of detection was due to exocytosis or digestion. Several ways of explaining the release of protein into the extracellular fluid have already been investigated [13,14], including those involving exosomes [15,16]. In addition, the possible digestion of protein inside lysosomes should be explored given the recently published result that primary cortical neurons show an increased Tau secretion when lysosomal function is reduced [17]. In order to confirm these preliminary results, the same experiments should be performed with the Tau proteins produced in eukaryotic cells. Indeed, bacteria do not possess the sophisticated mechanisms present in eukaryotes that would allow post-translational modifications and appropriate folding [18]. Such a technique would also avoid the contamination of purified proteins with lipopolysaccharides (LPS). The mechanisms involved in Tau internalization and processing must be further explored as well as the possible synaptic transmission of the highly phosphorylated Tau. We detected Tau in a small amount as dimers and trimers only in non-denaturing conditions, which suggests that they were converted to monomers during sample processing for western blot analysis. However, low and highly phosphorylated forms showed similar profiles. These results suggest that an uptake of dimers or trimers is not to be excluded but the difference of quantity needed for the internalization of low (15 µg) and highly (1.5 µg) phosphorylated Tau is more likely due to the phosphorylation state of the protein than to different forms present in the samples.

In conclusion, these first results underline that Neuro2A and primary cortical cells are capable of taking up small species of Tau protein more readily when they have been highly phosphorylated, which could constitute the earliest stage of the spread of Alzheimer’s disease within the brain.

## 4. Materials and Methods

### 4.1. Full-Length Recombinant Tau Protein (1-441)

Proteins were a generous gift from Neurhumab (Liège, Belgium). Human 2N4R Tau, the longest isoform, was expressed in a pET28a expression system (Novagen-Merck Millipore, Darmstadt, Germany) in *Escherichia* *coli* KRX strain (Promega, Madison, WI, USA) in 2 L of Terrific Broth medium (Sigma-Aldrich, St Louis, MO, USA) supplemented with 50 µg/mL kanamycin at 37 °C. Once the optical density at 600 nm (OD_600_) reached 1 unit, the temperature was lowered at 25 °C followed by an induction with 0.1% rhamnose and 2 mM IPTG. At OD_600_ = 4, the cells were harvested and disrupted on a French press. The soluble fraction was recovered by centrifugation at 12,000× *g* during 30 min at 4 °C and filtered on 0.45 µm. This fraction was heated to 75 °C in a water bath for 25 min to precipitate irrelevant proteins. Then, another centrifugation was performed as previously described. The supernatant containing the soluble fraction of the human 2N4R Tau was purified by immobilized metal ion affinity chromatography (IMAC) using a nickel-charged resin. IMAC purification was performed as follows: soluble fraction was dialyzed twice for 4 h against 50 mM Tris (pH 8) and the same buffer was used to equilibrate the HiTrap IMAC FF (GE Healthcare, Pittsburgh, PA, USA). Then, the resin was washed with 30 column volume (CV) of 50 mM Tris (pH 8); 10 CV of 50 mM Tris (pH 8), 10 mM imidazole; 10 CV of 50 mM Tris (pH 8), 2 M NaCl and finally, a last washing step with 10 CV of 50 mM Tris (pH 8), 0.1% Triton™ X-114 (Sigma-Aldrich, St Louis, MO, USA). Proteins were eluted in one step with 250 mM imidazole in 50 mM Tris (pH 8). LPS were eliminated by a treatment with 0.05% Triton™ X-114 (v/v) at 4 °C during 30 min and then at 25 °C during 15 min. A centrifugation at 2500× *g* at 25 °C was performed to recover the supernatant containing the detoxified protein, which was further filtered on 0.22 µm. Samples were sent to Lonza (Verviers, Belgium) for validation of LPS levels. Concentrations were obtained by measuring absorbance of purified proteins at 205 nm. The protein was supplied in a low phosphorylated form as well as in a highly phosphorylated form thanks to a co-expression with GSK3β, a serine/threonine kinase. In both cases, the two Tau proteins were fused to a C-terminal His-tag (6× His) to allow their detection by immunocytochemistry (Figure 1A).

### 4.2. Determination of the Protein Sequence and Phosphorylation Sites by Mass Spectrometry

The phosphorylation sites were identified as previously described [19]. Briefly, tryptic peptides data were analyzed by online nanoflow high-performance liquid chromatography tandem mass spectrometry (HPLC-MS/MS) using a NanoLC-Ultra 2D HPLC (Eksigent, Nieuwerkerk aan den Ijssel, The Netherlands) coupled to a TripleTOF^®^ 5600 mass spectrometer (AB Sciex, Nieuwerkerk aan den Ijssel, The Netherlands). After injection, peptide mixtures were transferred onto the analytical C18-nanocapillary HPLC column (C18 Acclaim PepMap100, 75 μm I.D.Å~15 cm, 3 μm particle size, 100 Å pore size, Dionex, ThermoScientific, Erembodegem-Aalst, Belgium) and eluted at a flow rate of 300 nL/min using the following gradient: at 3% solvent B in A (from 0 to 13 min), 3%–7% solvent B in A (from 13 to 16 min), 7%–25% solvent B in A (from 16 to 48 min), 25%–40% solvent B in A (from 48 to 65 min), 40%–90% solvent B in A (from 65 to 75 min) and at 90% solvent B in A (from 75 min to 85 min), with a total runtime of 120 min including mobile phase equilibration. Solvents were prepared as follows, mobile phase A: 2% acetonitrile/98% of 0.1% formic acid (v/v) in water, and mobile phase B: 98% acetonitrile/2% of 0.1% formic acid (v/v) in water. The mass spectrometer was operated in positive ion mode with an electrospray voltage of 2300 V. The mass spectrometry (MS) data acquisition cycle consisted of a single MS scan with a range of 400 to 1250 *m*/*z* followed by MS/MS scans from 100 to 1800 *m*/*z* of the most abundant ions detected in the preceding MS scan. The number of MS/MS scans per MS scan was limited to 20. Peptides and phosphopeptides were identified from the tandem mass spectra using ProteinPilot^TM^ software and the Paragon™ algorithm (version 4.5.0.0,1654) (AB SCIEX beta 4.5, revision 1656, Nieuwerkerk aan den Ijssel, The Netherlands) with phosphorylation emphasis and a thorough protein identification (ID) search effort specified in the search parameters. The general sample parameters were used: trypsin digestion, cysteine alkylation set to iodoacetamide. All data files were searched using the Uniprot_Trembl_112013 containing the sequence of the recombinant protein. For database searches, a cut-off peptide confidence value of 99 was used and phosphorylation site assignments were checked manually. The ratios of the signal intensities of peptide versus phosphopeptide were estimated for each identified phosphopeptide as described by Steen and collaborators, 2006 [20].

### 4.3. Western Blot Analysis

For sample analysis, 5 µL of PageRuler^TM^ Plus Prestained Protein Ladder (Thermo Fisher Scientific, Vilnius, Lithuania) and 500 ng of low and highly phosphorylated Tau proteins were loaded for each condition on a 4%–20% polyacrylamide gel (Mini-PROTEAN^®^ TGX™, Bio-Rad, Hercules, CA, USA), and allowed to migrate during 1 h at 50 mA in 1× Tris-Glycine-SDS buffer (Bio-Rad). Boiling and β-mercaptoethanol were avoided to preserve multimeric complexes. The transfer was performed at 4 °C for 1 h 45 min and 260 mA (1× Tris-Glycine buffer (Bio-Rad) with 20% methanol) on a 0.45 µm nitrocellulose membrane (Amersham^TM^ Proton^TM^, GE Healthcare, Berlin, Germany) which was incubated overnight with the corresponding primary antibodies diluted in Tris buffered saline (TBS)-Tween-20 0.05% (Sigma-Aldrich, St Louis, MO, USA) containing 5% bovine serum albumin (BSA): rabbit anti-phosphoT231 antibody 1:1000 (Abcam, Cambridge, UK), mouse anti-Tau antibody 1:1000 (Invitrogen, Carlsbad, CA, USA) and mouse anti-His-tag antibody 1:2000 (Invitrogen). Incubation with secondary antibodies was carried out for 1 h, either with an anti-mouse or an anti-rabbit (1:10,000) from sheep coupled to horseradish peroxidase (HRP) (GE Healthcare Biosciences, Uppsala, Sweden) and diluted in TBS-Tween-20 0.05%. All washing steps were accomplished in TBS-Tween-20 0.05%.

### 4.4. BN-PAGE

This technique was used to maintain the native conformation of proteins and separate them depending on their size to distinguish different forms of aggregates with a low influence of their isoelectric point [21]. The migration was performed following manufacturer’s instructions (NativePAGE™ Novex™ 4%–16% Bis-Tris Protein Gels Protocol, Thermo Fisher Scientific). Then, Western blot was performed as described above, with mouse anti-Tau primary antibody 1:1000 (Invitrogen) targeted by anti-mouse secondary antibody (1:10,000) from sheep coupled to HRP (GE Healthcare Biosciences, Uppsala, Sweden).

### 4.5. Rat Primary Embryonic Cortical Cultures

Glass coverslips were pre-coated with 0.1 mg/mL of poly-l-lysine for 2 h (Sigma-Aldrich, St Louis, MO, USA). Primary cortical cultures were obtained from Wistar rat embryos at E19 and plated in a 24-well plate at a density of 100,000 cells/well. They were cultivated for 13 days at 37 °C and 5% CO_2_ in Neurobasal Medium supplemented with 2% B27^®^ (Thermo Fisher Scientific), 1% foetal bovine serum (FBS; Gibco, New York, NY, USA), 0.5 mM l-glutamine (Gibco, Paisley, UK) and 50 µg/mL antibiotics (penicillin and streptomycin; Invitrogen). The next day, the medium was totally changed and then, twice a week by 50%. All animal procedures were carried out according to European Communities Council guidelines for the care and use of animals in research and with the agreement of the local ethics committee.

### 4.6. Neuro2A Cultures

Neuro2A cells were plated in 24-well plates at a density of 50,000 cells/well in Dulbecco’s modified Eagle medium (DMEM)-Glutamax™ (Thermo Fisher Scientific) supplemented with 6% FBS and 50 µg/mL gentamicin (Invitrogen) for one night to acquire an approximate density of 100,000 cells/well the next day. They were also kept at 37 °C and 5% CO_2_. In the morning, the medium was replaced by a differentiation medium (DMEM-Glutamax™, 50 µg/mL gentamicin, 0.1% BSA (Acros Organics, Illkirch, France) to allow the formation of neurites and renewed after 24 h.

### 4.7. Addition of Proteins in the Extracellular Fluid

**Figure A6:**
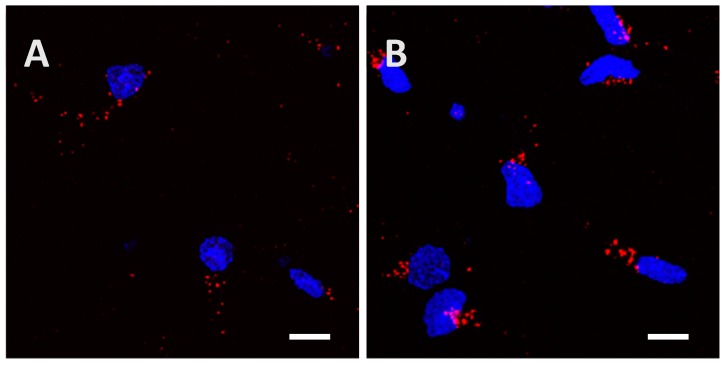
Low phosphorylated (15 µg; **A**) and highly phosphorylated Tau proteins (1.5 µg; **B**) were visible inside cortical cells after 24 h of incubation even when a washing with 0.05% of trypsin for 1 min was performed. Trypsin was used to remove potential proteins attached to the extracellular matrix or the cytoplasmic membrane. Scale bars, 10 µm.

Proteins were added at day in vitro (DIV) 13 for cortical cultures and after 48 h of differentiation for Neuro2A cultures. They were placed in the appropriate medium with 1.5 µg, 4.5 µg or 15 µg for Tau protein and 1.5 µg for the hyperphosphorylated Tau protein. In both culture types, proteins were maintained for 8 h, 24 h and 48 h. However, Neuro2A cells were incubated 8 h with proteins prior to a medium change containing no protein for 48 h while cortical cells were incubated 8 h or 24 h before medium change for additional 24 h (Figure A5; Figure 2 and Figure 3). A washing with 0.05% trypsin for 1 min was also made after 24 h of incubation as a control (Figure A6).

### 4.8. Detection of Tau Proteins by Immunocytochemistry

Fixation was performed with 4% paraformaldehyde (Sigma-Aldrich, Darmstadt, Germany) for 10 min at 4 °C followed by 5 min at room temperature. Between each step described below, the permeabilization and washings of membranes were achieved three times in phosphate buffered saline (PBS)-Triton™ X-100 0.2% (Sigma-Aldrich, St Louis, MO, USA). Non-specific sites were blocked by treating cells with a blocking solution (0.05% casein (Sigma-Aldrich, St Louis, MO, USA), 0.05 M NH_4_Cl in PBS) for 30 min. Then, primary antibodies were diluted in the blocking solution using a dilution of 1:500 for the mouse monoclonal anti-His-tag antibody (Invitrogen) to detect Tau proteins and a dilution of 1:400 for the rabbit polyclonal anti-MAP2 antibody (Millipore, Overijse, Belgium), which detected microtubules of neuronal dendrites by targeting MAP2. Coverslips were incubated with primary antibodies for 90 min before exposing them for 30 min with appropriate secondary antibodies also diluted in blocking solution (anti-mouse AlexaFluor^®^ 594 from goat, 1:500 (Invitrogen), and anti-rabbit AlexaFluor^®^ 488 from goat, 1:500 (Invitrogen). Coverslips were analyzed by confocal imaging (Olympic Fluoview FV1000, Olympus, Berchem, Belgium). All images were obtained at a magnification of 60× by Z-projection with a step size of 0.46 µm between each captured image but a varying number of slices depending on cell thickness.

### 4.9. Evaluation of the Anti-His-Tag Antibody Affinity for Proteins by ELISA

Proteins were immobilized in duplicate with a quantity of 1 µg overnight at 4 °C in 96-well plates thanks to a 0.1 M sterile bicarbonate buffer (pH 8.6). Incubations were performed at room temperature for 1 h and washings were made 3 times between each step by a PBS-Tween-20 0.5% buffer. Non-specific sites were blocked with Protein-Free Blocking Buffer at pH 7.4 (Thermo Fisher Scientific). Then, the mouse anti-His-tag antibody (1:200) was diluted in PBS-Tween-20 0.1% buffer at pH 7.8 and placed into wells to be targeted by a biotinylated anti-mouse secondary antibody from goat (VectorLabs, Burlingame, Canada) diluted in the same buffer (1:300). We used the Avidin-Biotin Complex (ABC) kit (VectorLabs) which comprised the HRP at 4 °C for 1 h. Finally, the substrate consisting of ABTS (pH 4) supplemented with 1.7 µL of 30% hydrogen peroxide was added and maintained for 10 min to obtain a green coloration. Optical densities were measured by a spectrophotometer at a wavelength of 405 nm (Stat-Fax 2100, Awareness Technology, Inc., Perchtoldsdorf, Austria).

## References

[1] Iqbal K., Liu F., Gong C.X., Grundke-Iqbal I. (2010). Tau in Alzheimer disease and related tauopathies. Curr. Alzheimer Res..

[2] Liu L., Drouet V., Wu J.W., Witter M.P., Small S.A., Clelland C., Duff K. (2012). Trans-synaptic spread of Tau pathology in vivo. PLoS ONE.

[3] Clavaguera F., Bolmont T., Crowther R.A., Abramowski D., Frank S., Probst A., Fraser G., Stalder A.K., Beibel M., Staufenbiel M. (2009). Transmission and spreading of tauopathy in transgenic mouse brain. Nat. Cell Biol..

[4] De Calignon A., Polydoro M., Suárez-Calvet M., William C., Adamowicz D.H., Kopeikina K.J., Pitstick R., Sahara N., Ashe K.H., Carlson G.A. (2012). Propagation of Tau pathology in a model of early Alzheimer’s disease. Neuron.

[5] Calafate S., Buist A., Miskiewicz K., Vijayan V., Daneels G., De Strooper B., de Wit J., Verstreken P., Moechars D. (2015). Synaptic contacts enhance cell-to-cell Tau pathology propagation. Cell Rep..

[6] Kfoury N., Holmes B.B., Jiang H., Holtzman D.M., Diamond M.I. (2012). Trans-cellular propagation of Tau aggregation by fibrillar species. J. Biol. Chem..

[7] Frost B., Jacks R.L., Diamond M.I. (2009). Propagation of Tau misfolding from the outside to the inside of a cell. J. Biol. Chem..

[8] Srögren M., Davidsson P., Tullberg M., Minthon L., Wallin A., Wikkelso C., Granrus A.-K., Vanderstichele H., Vanmechelen E., Blennow K. (2001). Both total and phosphorylated Tau are increased in Alzheimer’s disease. J. Neurol. Neurosurg. Psychiatry.

[9] Tai H.C., Wang B.Y., Serrano-Pozo A., Frosch M.P., Spires-Jones T.L., Hyman B.T. (2014). Frequent and symmetric deposition of misfolded Tau oligomers within presynaptic and postsynaptic terminals in Alzheimer’s disease. Acta Neuropathol. Commun..

[10] Michel C.H., Kumar S., Pinotsi D., Tunnacliffe A., George-Hyslop P.S., Mandelkow E., Mandelkow E.-M., Kaminski C.F., Schierle G.S.K. (2014). Extracellular monomeric Tau protein is sufficient to initiate the spread of Tau protein pathology. J. Biol. Chem..

[11] Wu J.W., Herman M., Liu L., Simoes S., Acker C., Figueroa H., Steinberg J.I., Margittai M., Kayed R., Zurzolo C. (2012). Small misfolded Tau species are internalized via bulk endocytosis and anterogradely and retrogradely transported in neurons. J. Biol. Chem..

[12] Dujardin S., Lécolle K., Caillierez R., Bégard S., Zommer N., Lachaud C., Carrier S., Dufour N., Aurégan G., Winderickx J. (2014). Neuron-to-neuron wild-type Tau protein transfer through a trans-synaptic mechanism: Relevance to sporadic tauopathies. Acta Neuropathol. Commun..

[13] Mohamed N.V., Herrou T., Plouffe V., Piperno N., Leclerc N. (2013). Spreading of Tau pathology in Alzheimer’s disease by cell-to-cell transmission. Eur. J. Neurosci..

[14] Dujardin S., Bégard S., Caillierez R., Lachaud C., Delattre L., Carrier S., Loyens A., Galas M.-C., Bousset L., Melki R. (2014). Ectosomes: a new mechanism for non-exosomal secretion of Tau protein. PLoS ONE.

[15] Chivet M., Javalet C., Laulagnier K., Blot B., Hemming F.J., Sadoul R. (2014). Exosomes secreted by cortical neurons upon glutamatergic synapse activation specifically interact with neurons. J. Extracell. Vesicles.

[16] Asaï H., Ikezu S., Tsunoda S., Medalla M., Luebke J., Haydar T., Wolozin B., Butovsky O., Kégler S., Ikezu T. (2015). Depletion of microglia and inhibition of exosome synthesis halt Tau propagation. Nat. Neurosci..

[17] Mohamed N.V., Plouffe V., Rémillard-Labrosse G., Planel E., Leclerc N. (2014). Starvation and inhibition of lysosomal function increased Tau secretion by primary cortical cultures. Nature.

[18] Overton T.W. (2014). Recombinant protein production in bacterial hosts. Drug Discov. Today.

[19] Makhmoudova A., Williams D., Brewer D., Massey S., Patterson J., Silva A., Vassall K.A., Liu F., Subedi S., Harauz G. (2014). Identification of multiple phosphorylation sites on maize endosperm starch branching enzyme IIb, a key enzyme in amylopectin biosynthesis. J. Biol. Chem..

[20] Steen H., Jebanathirajah J.A., Rush J., Morrice N., Kirschner M.W. (2006). Phosphorylation analysis by mass spectrometry: Myths, facts, and the consequences for qualitative and quantitative measurements. Mol. Cell. Proteom..

[21] Schägger H., Von Jagow G. (1991). Blue native electrophoresis for isolation of membrane protein complexes in enzymatically active form. Anal. Biochem..

